# ASO Author Reflections: From Feasibility to Implementation: Establishing Indocyanine Green as a Single-Tracer Strategy for Sentinel Lymph Node Biopsy in Breast Cancer

**DOI:** 10.1245/s10434-026-20444-3

**Published:** 2026-08-19

**Authors:** Vassilis Pitsinis, Nadia Khitab, Lee Jordan, Alessio Vinci, Rahul Kanitkar, Emad Elseedawy, Jennifer Smith

**Affiliations:** 1 NHS Tayside, Breast Surgery, Dundee, UK; 2 NHS Tayside, Pathology, University of Dundee, Dundee, UK

## Past

Sentinel lymph node biopsy has transformed axillary staging in early breast cancer by providing accurate nodal assessment while substantially reducing the morbidity associated with axillary lymph node dissection. Nevertheless, conventional sentinel node mapping continues to rely on radioisotope, often combined with blue dye, creating dependence on nuclear medicine facilities, regulatory requirements, additional costs, and logistical complexity and significant staining and allergic risk potential. These limitations have encouraged the development of alternative non-radioactive approaches, including superparamagnetic iron oxide tracers. Although such methods have demonstrated clinical utility, their wider adoption may be limited by factors, including skin staining, magnetic resonance imaging artefact, the need for dedicated detection equipment, and potential compatibility considerations in patients with certain implanted medical devices. Since the first reports of indocyanine green fluorescence-guided lymphatic mapping, numerous studies have demonstrated promising detection rates, while subsequent meta-analyses have suggested that ICG achieves performance comparable with conventional tracer techniques. However, much of the available evidence has arisen from retrospective studies, relatively small cohorts, or mixed-tracer protocols, leaving uncertainty regarding the reliability of ICG when used as the sole mapping agent during routine clinical practice.^1–3^

## Present

Our prospective UK cohort provides real-world evidence supporting the use of ICG as a standalone tracer for sentinel lymph node biopsy. Across 300 consecutive procedures, ICG achieved a procedural detection rate of 99.7% and a fluorescent nodal detection rate of 97.7%, with performance remaining consistent across a broad spectrum of patient age, body mass index, tumour size and operative approach. Importantly, procedures were undertaken by surgeons with varying levels of experience in fluorescence-guided surgery, demonstrating that excellent detection rates can be maintained beyond highly specialised centres. These findings complement the multicentre randomised INFLUENCE trial by demonstrating that the excellent performance of ICG is reproducible during routine implementation in everyday breast surgical practice.^4,5^

## Future

Future research should move beyond demonstrating technical feasibility towards defining the optimal role of fluorescence-guided surgery within modern breast cancer care. Large multicentre implementation studies, health-economic evaluations, and long-term oncological outcome analyses will further clarify the clinical and economic benefits of replacing radioisotope-based mapping. As fluorescence imaging technology becomes increasingly available and surgeons gain greater familiarity with near-infrared guidance, ICG-only sentinel lymph node biopsy has the potential to simplify surgical pathways, improve accessibility, and facilitate wider global adoption of radiation-free axillary staging.

## References

[1] Kitai T, Inomoto T, Miwa M, Shikayama T. Fluorescence navigation with indocyanine green for detecting sentinel lymph nodes in breast cancer. *Breast Cancer*. 2005;12(3):211–5. 10.2325/jbcs.12.211 16110291

[2] He K, Chi C, Kou D, Huang Y, Zhang Y, Li S. Indocyanine green fluorescence versus radioisotope for sentinel lymph node biopsy in breast cancer: systematic review and meta-analysis. *Breast Cancer Res Treat*. 2021;186(3):523–34.

[3] Goldhawk-White P, White KP, Doeksen A, McGoldrick C, Neligan PC, Newman MI, Pitsinis V, Schols RM. Fluorescence imaging with indocyanine green during breast cancer surgery and postmastectomy breast reconstruction: a double systematic review and cost analysis. *Surgery*. 2026;192:110052. 10.1016/j.surg.2025.110052 41570662

[4] Pitsinis V, Kanitkar R, Vinci A, Choong WL, Benson JR. Results of a prospective randomized multicenter study comparing indocyanine green fluorescence combined with a standard tracer versus indocyanine green alone for sentinel lymph node biopsy in early breast cancer: the INFLUENCE trial. *Ann Surg Oncol*. 2024;31(13):8848–55. 10.1245/s10434-024-16176-x PMC11549146 39266795

[5] Pitsinis V, Khitab N, Jordan L, Vinci A, Kanitkar R, Elseedawy E, Smith J. ICG-only sentinel lymph node biopsy in breast cancer: a prospective UK cohort demonstrating consistent detection across patient and tumour characteristics—a follow-on to the INFLUENCE trial. *Ann Surg Oncol*. 2026. 10.1245/s10434-026-20316-w. PMC13675259 42527803

